# Long-Term Impact of Sustained Knowledge, Confidence, and Clinical Application Following a First-Year Student Pharmacist Diabetes Self-Care Education Program

**DOI:** 10.3390/pharmacy13020042

**Published:** 2025-03-11

**Authors:** Candis M. Morello, Eduardo S. Fricovsky

**Affiliations:** Skaggs School of Pharmacy and Pharmaceutical Sciences, University of California San Diego, La Jolla, CA 92093, USA; esfricovsky@health.ucsd.edu

**Keywords:** diabetes education, pharmacy education, diabetes self-care, student pharmacist, pharmacist

## Abstract

With diabetes reaching epidemic proportions globally, it is imperative to increase the number of providers equipped to screen, educate, and help patients achieve glycemic control. This study evaluated the long-term results of student pharmacists attending a first-year Diabetes Self-Care Education Program (DSEP) by measuring knowledge retention, confidence, and clinical applicability of skills learned over time. The DSEP, integrated into the early pharmacy curriculum, is a 9-h training program made up of interactive lectures, glucose monitoring assignments, and active-learning workshops. Following DSEP training, two cohorts of first-year student pharmacists were surveyed annually for 3 and 5 years to assess knowledge retention, confidence, and clinical use of the DSEP content in their practice sites. By the year 1 follow-up survey, the response rate from the pre-survey period for cohort 1 was 88% and 78% for cohort 2; over time, the response rate decreased. For the long-term follow-up surveys, cohort 1 (5 years) and cohort 2 (3 years) demonstrated overall significantly improved sustained knowledge of diabetes (48% higher average test score, *p* < 0.001), perceived confidence, and clinical ability (over 60% and 76% increases from baseline, *p* < 0.001). Within 12 months of completing the DSEP, about two-thirds of students applied their training to assist patients with diabetes and their caregivers. Long-term, participants in both cohorts reported educating and screening over 22,000 patients with diabetes and caregivers in multiple clinical settings over 3 years and 5 years, respectively, following DSEP training. The positive impact of improved knowledge, confidence, and clinical applicability of the DSEP training obtained by student pharmacists was sustained for 3 or more years, impacting thousands of patients with diabetes and caregivers. Considering the growing global diabetes epidemic, pharmacy schools around the world should consider implementing an early DSEP program.

## 1. Introduction

Diabetes prevalence has grown to epidemic proportions. With over one-third of the United States (U.S.) population either diagnosed with diabetes or at risk of developing this metabolic disorder and approximately 527 million adults globally living with diabetes, it is vital to increase the number of providers equipped to screen, educate, and help these patients achieve glycemic control [1,2]. Long-term complications of diabetes are costly and can be mitigated or delayed by managing blood glucose, blood pressure, and cholesterol levels, in addition to offering diabetes-specific education and encouragement for preventive care [3,4,5,6]. Patients with diabetes who are well-informed about diabetes self-care and their medications tend to achieve better glycemic control [7]. Pharmacists are ideally situated in community, clinical, and inpatient settings to leverage their specialized knowledge and skills expertise in medications, communication, and comprehensive medication management to enhance care and education for patients with diabetes and their caregivers [8,9,10,11]. As indicated in the latest American Diabetes Standards of Care guidelines, creating a patient-centered care plan is essential and requires a multipronged approach that involves more than treatment with medications [12]. Moreover, pharmacists’ involvement in non-pharmacological self-care interventions can further improve patients’ understanding of diabetes and glycemic control, which in turn may prevent or delay diabetes complications. Key self-care interventions include overall diabetes education, preventive care for eyes, feet, and skin, vaccinations, nutritional guidance, physical activity education, risk-factor analysis, glucose monitoring, insulin and injection administration training, and hypoglycemia management [12].

All accredited pharmacy programs in the U.S. are required to provide self-care education, and students must engage in introductory practice experiences and internships from their first year [13,14]; however, no one has evaluated the impact of having this education in the first year of pharmacy school. Implementing a comprehensive diabetes self-care training program early in their education could better prepare student pharmacists to educate patients and contribute to the growing need for healthcare providers to address the diabetes crisis. While some studies have explored the effectiveness of diabetes education programs, most have focused on an offered elective or advanced courses offered in later years of pharmacy school or contents that did not include comprehensive self-care in the first year [15,16,17,18,19]. At the University of California (UC) San Diego Skaggs School of Pharmacy and Pharmaceutical Sciences (SSPPS), an innovative first-year Diabetes Self-care Education Program (DSEP) was developed and implemented within the Pharmacy Practice Course. The course description and impact of pre-, post-, and 9-month follow-up outcomes were previously published [20]. While this study described the comprehensive DSEP training, provided the survey tools used, and concluded that the DSEP training significantly improved student pharmacists’ knowledge and confidence in diabetes self-care education and that participants immediately used their skills in the short term, it did not evaluate long-term clinical applicability, use, and impact. This follow-up study explored the clinical use, application, and impact of cohorts 1 and 2 in the years following DSEP training at UC San Diego SSPPS for 3 years and 5 years (cohorts 2 and 1, respectively). The objectives were to assess: (1) the effectiveness of the course by measuring the diabetes knowledge acquired and student pharmacist perception of confidence in providing education, (2) the retention of this knowledge over time, and (3) the application and use of this knowledge in clinical settings. The aim was to demonstrate that early participation in a diabetes self-care education program benefits student pharmacists and enhances their ability to educate patients with diabetes and caregivers, with the expectation that this training will improve both short-term and long-term retention of diabetes knowledge and increase confidence in patient education as well as evaluate the impact.

## 2. Methods and DSEP Training Design

As previously described [20], the 9-h first-year student pharmacist DSEP training consisted of 3 sections: (1) lectures, (2) a home glucose monitor assignment, and (3) active-learning workshops on self-care topics. The program’s curricular content was adapted from the diabetes chapter that existed in the Handbook of Nonprescription Drugs: An Interactive Approach to Self-Care along with pertinent content from the Association of Diabetes Care and Education (ADCES) Core Curriculum, with current content found in ADCES The Art of Science of Diabetes Care and Education [21,22]. The interactive lecture component consisted of a two-hour session on nonprescription diabetes management, designed to equip student pharmacists with a comprehensive understanding of diabetes care. The first hour was dedicated to an overview of diabetes, screening, and preventive measures. Key topics included the nonpharmacologic interventions (such as preventive care, medication nutrition therapy, and physical activity, immunizations), and the pharmacist’s role in diabetes screening. The preventive care discussion emphasized recommended vaccinations and the importance of oral, dental, skin, and eye care. The second hour concentrated on insulin therapy in diabetes management, covering insulin pharmacology, various insulin products, and the pharmacokinetic profiles of different insulin regimens. Additionally, the role of pharmacists in educating, training, and supporting patients on insulin therapy was explored.

The second part involved a home glucose monitoring assignment, aimed at giving student pharmacists hands-on experience with self-monitoring blood glucose. Students were instructed to read the manual, set up the device, and perform at least five blood glucose measurements (either before meals, two hours after meals, or at bedtime) over two weeks, recording their findings in the logbook. This self-monitoring exercise allowed students to gain empathy and a deeper understanding of the daily tasks required of patients with diabetes. Upon completing the assignment, the student pharmacists shared their experiences and compared glucose monitors during a diabetes workshop, identifying features that might be beneficial for different patient groups. This task allowed students to learn the techniques of self-monitoring, enabling them to teach fellow students during the workshop.

The diabetes workshops consisted of two 2-h sessions. The first session included a combined lecture, and “hands-on” exercises focused on insulin administration, both vial/syringe and pen use, factors influencing insulin absorption rates and hypoglycemia prevention and treatment using normal saline as a substitute for insulin, students practiced preparing “insulin” doses and performing subcutaneous injections on themselves. The session also covered the differences between available glucose monitors and reviewed the results from the home glucose monitoring assignment. At the end of the session, students applied their learning to a patient case study.

The second workshop, focused on diabetes foot care, combined a lecture with practical exercises on the causes, symptoms, and treatment options for diabetic foot disorders, including both pharmacologic and nonpharmacologic approaches. Students also learned how to educate patients on general foot care guidelines, how to conduct a comprehensive foot examination, and how to apply their knowledge to a patient case. All student pharmacists performed foot exams on their peers using a “look, listen, and feel” method, palpating pedal pulses, and testing for pedal sensation with 10-g monofilaments.

Three survey tools were developed and published in our previous study [20], and were used to measure diabetes knowledge gained, student pharmacist confidence in providing diabetes education, and how much they applied this education in real clinical practice. Within the surveys, a case-based knowledge test evaluated the knowledge gained and the application and integration of information. This test consists of 16 multiple-choice questions focused on challenges faced by patients with type 1 or type 2 diabetes. In addition to the case-based knowledge test, the survey instruments included 19 multipart questions related to overall knowledge and confidence in performing finger-stick glucose tests, insulin injections, and foot care. Most of the survey questions used a 5-point Likert scale, ranging from 1 to 5, with 1 indicating the lowest response (e.g., poor, not at all, or strongly disagree) and 5 representing the highest response (e.g., excellent, extremely, or strongly agree).

The follow-up survey included all content from the post-program survey, with the addition of questions assessing the clinical application of the knowledge gained. This survey contained 41 additional questions regarding exposure to clinical practice opportunities since the program’s completion, the relevance of the knowledge to practice, and the application of learned skills in real-world settings. It was administered one year after the baseline pre-program survey, allowing student pharmacists time to apply their knowledge during their pharmacy internships in the summer following the program.

Follow-up surveys were sent to the Class of 2011 (cohort 1) and Class of 2013 (cohort 2) student pharmacists at the UCSD SSPPS who had previously participated in the DSEP training. The cohorts were followed annually for 3 years and 5 years (cohort 2 and cohort 1, respectively). This study was reviewed and approved by the UC San Diego Human Research Protections Program. Informed consent was obtained from all study subjects. Participants were entered into a drawing at each survey time point for a $30 gift card for the UC San Diego Book Store.

Descriptive statistics were used to summarize the findings. Friedman tests assessed within-subject differences at the three time points. Wilcoxon signed-rank tests were used for pairwise comparisons within each individual cohort, and Wilcoxon rank-sum tests compared results between the two cohorts, with *p* < 0.05 in two-tailed tests considered significant.

## 3. Results

### 3.1. Demographics

Baseline demographics for cohort 1 and cohort 2 were indicative of the UC San Diego SSPPS student pharmacist population. Most student participants were female (78%) with a mean age of 24 years old. None reported having diabetes; however, 21 subjects (36%) reported having a family member with either type 1 or type 2 diabetes. Only 1 (1.7%) subject reported having experienced prior formal diabetes training [20].

Of the sixty first-year pharmacy students enrolled in the Pharmacy Practice Course SPPS201, 59 enrolled in the study as cohort 1 and completed the pre-survey. Fifty-eight of the 59 (98%) enrolled subjects completed the post-survey, 54 (92%) completed the follow-up survey at 9 months, 52 (88%) at 12 months, 26 (44%) at 24 months, 18 (31%) at 36 months, 4 (7%) at 48 months, and 11 (19%) at 60 months post-survey. For cohort 2, 60 participants enrolled and 60 (100%) completed the pre-survey and post-survey; 48 (80%) completed the follow-up survey at 9 months, 47 (78%) at 12 months, 22 (37%) at 24 months, and 12 (20%) at 36 months (Table 1). Due to lack of funding, no further follow-up surveys were done.

### 3.2. Overall Confidence and Ability to Provide Diabetes Care

**Figure 1:**
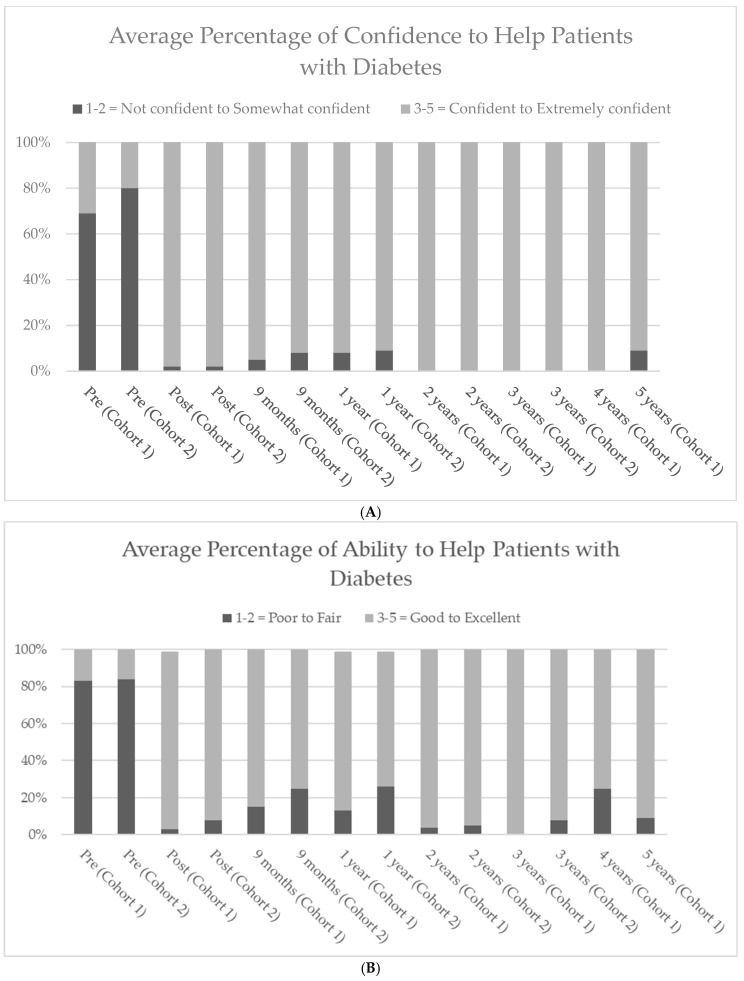
(**A**) Depicts the average percentage of student pharmacists reported confidence in helping patients with diabetes over the study period for cohort 1 (5 years) and cohort 2 (3 years) comparing confident to extremely confident to not confident and somewhat confident. For both cohorts, compared to pre-test values and each subsequent endpoint, confidence in helping patients with diabetes significantly improved and sustained over time. (**B**) Depicts the average percentage of student pharmacists reported ability in helping patients with diabetes over the study period for cohort 1 (5 years) and cohort 2 (3 years) comparing good to excellent and poor to fair. For both cohorts, compared to pre-test values and each subsequent endpoint, ability in helping patients with diabetes significantly improved and sustained over time.

As previously reported, a comparison of the pre- and post-survey scores in cohort 1 and cohort 2 showed significant increases in the student’s confidence and ability after participating in the DSEP (*p* < 0.001) [20]. Prior to the DSEP, 2% of subjects in cohort 1 and 7% in cohort 2 rated their overall confidence in helping patients with diabetes as very or extremely confident compared to 64% in cohort 1 and cohort 2 after the DSEP. Prior to the DSEP, 3% in cohort 1 and 8% in cohort 2 rated their overall ability to help patients with diabetes as very or extremely good, compared to 58% in cohort 1 and 56% in cohort 2, after the DSEP (Figure 1A). Subsequent follow-up surveys also show significant confidence and ability to help patients with diabetes (*p* < 0.01) compared with pre-surveys (Figure 1A,B). Prior to the DSEP, the percentage of confidence ranging from confident to very confident average between cohort 1 and cohort 2 was 25% and the ability ranging from good to excellent average was 17%. After the DSEP training cohort 1 and cohort 2 follow-up surveys, the average confidence in helping patients as confident to very confident average was 96%. Similarly, the average ability to help patients with diabetes ranging from good to excellent was high at 86%. Due to the low number of respondents (N = 4) in cohort 1, year 4, caution is needed in interpreting the results. Despite this limitation, statistically significant improvements were observed in student confidence in areas such as performing and teaching the finger-stick test, insulin injection, managing low blood glucose, and conducting diabetic foot exams. However, the lack of significant changes in some areas may be attributed to the small sample size, which limits the ability to detect differences in other areas.

### 3.3. Overall Diabetes Knowledge and Confidence with Monitors, Insulin Use, and Foot Exams

Initially, in pre-DSEP training, most of the diabetes knowledge was poor to fair, with the lowest in areas of insulin therapy, glucose monitoring, and diabetic foot exams. Notably, by year 3 (cohort 2) and year 5 (cohort 1), knowledge was reported as >80% sustained in diabetes as a disease, risk factors, complications, differences between type 1 and type 2 diabetes, signs and symptoms of diabetes, nutrition therapy, exercise guidelines, and insulin therapy. While glucose monitoring and diabetic foot exams achieved this high threshold (>80%) in cohort 2, in cohort 1, they still increased but were 72% and 54%, respectively (Table 1).

Similarly, following the DSEP training in evaluating confidence, participants reported that overall confidence and ability to help patients with diabetes in areas such as glucose monitoring and finger-sticks, insulin use, and diabetic foot exams were significantly improved and sustained (*p* < 0.001). In cohort 1, it was recorded that confidence to help patients with diabetes was improved and sustained by over 80% in all the categories over 5 years, except in the subcategory about teaching patients how to perform a diabetic foot exam on themselves, which was 73%. In cohort 2, confidence was high over 90% in all categories and was sustained over 3 years (Table 1).

### 3.4. Knowledge Assessment by Type 1 and Type 2 Diabetes Case-Based Knowledge Test

Prior report analysis of the pre- and post-survey knowledge test scores reflected significant increases in both the study subjects’ overall knowledge test as well as the individual multiple-choice case-based questions that involved patients with both type 1 and type 2 diabetes (Table 2). The percentage of questions answered correctly more than doubled when comparing pre-, post-, and follow-up survey scores (*p* < 0.001). The average case-based pre-survey percentage scores for both cohort 1 and cohort 2 were 32%, compared to 81% for cohort 1 at 5 years and 80% for cohort 2 at 3 years in the follow-up surveys. Both the post-survey and follow-up survey overall knowledge test scores were >48% higher than the baseline pre-survey scores (Table 2).

### 3.5. Clinical Applicability and Use of DSEP Knowledge

The follow-up survey contained additional questions assessing where, how much, and to whom the participants used their DSEP-acquired knowledge and skills at 9 months, then annually up to 5 years following training. Opportunities to assist patients with diabetes and their caregivers occurred mostly in large community-based pharmacies, as well as pharmacy clinics with more inpatient hospital pharmacy locations in the later years (Table 3). Following the DSEP training and by the end of their first year of pharmacy school (9-month follow-up survey), 61% of cohort 1 and 75% of cohort 2 of student pharmacists assisted both patients with diabetes and 17% and 18% (cohort 1 and cohort 2) assisted caregivers by using their knowledge in clinical settings (Table 4). This level of engagement and application was sustained throughout the follow-up years. Ninety percent of student pharmacists in cohort 1 reported assisting patients with diabetes at 5 years and 100% for cohort 2 at 3 years. Sixty-three percent of cohort 1 (5 years) said they assisted caregivers with patients with diabetes compared to 91% of cohort 2 (3 years) student pharmacists (Table 4). Throughout the study period, participants in both cohorts consistently reported that participating in the DSEP prepared them to educate patients with diabetes, increased their interest in diabetes, and plan to pursue further diabetes education as a result of their experience in the DSEP (Table 5).

## 4. Discussion

By receiving early comprehensive DSEP training, participants demonstrated that they attained better knowledge and confidence in areas of diabetes self-care to assist patients and caregivers, and the clinical application was sustained over time in varying clinical settings. Similar patterns were observed in both cohorts. Initially, in pre-DSEP training, the majority of diabetes knowledge was poor to fair, with the lowest in areas of insulin therapy, glucose monitoring, and diabetic foot exams. Notably, by year 3 (cohort 2) and year 5 (cohort 1), knowledge was reported as > 80% sustained in diabetes as a disease, risk factors, complications, differences between type 1 and type 2 diabetes, signs and symptoms of diabetes, nutrition therapy, exercise guidelines, and insulin therapy. While glucose monitoring and diabetic foot exams achieved this high threshold (> 80%) in cohort 2, they still increased in cohort 1 but were 72% and 54%, respectively. As students progressed through their curriculum, involving therapeutics offered in our third year, as well as IPPE and APPE opportunities, and then onto post-graduate training and jobs, especially for cohort 2, we would expect an increase in diabetes knowledge, which is definitely what we observed.

Similarly, in evaluating confidence, participants reported that overall confidence and ability to help patients with diabetes in all areas was significantly improved following the DSEP training and sustained over 3 years (cohort 2) and 5 years (cohort 1). Of note, insulin administration, glucose monitoring, and all areas of diabetic foot care were found to improve the most. This is important since many diabetes medications are administered by injection. Performing glucose monitoring, either by way of glucose monitoring or continuous glucose monitoring (CGM), is essential in the management of diabetes and the detection of hypoglycemia, and to help patients with diabetes achieve glycemic control. Glycemic control is associated with the prevention or delayed progression of long-term diabetes complications, especially microvascular complications such as retinopathy, nephropathy, and neuropathy [1,4,5]. For example, diabetes is the most common cause of non-traumatic amputations, which is associated with a high healthcare cost, not to mention the patient burden and higher risk of 5-year mortality following amputation. One major educational take-home message in the caring for the diabetic foot workshop is for student pharmacists to educate people with diabetes to inspect their feet daily (top, bottom, and in between toes) and to report any irregularities such as open wounds, erythema, or tinea, as this assists with early detection and treatment intervention.

One key outcome is that the participants in both cohorts for the entire study duration indicated that the DSEP prepared them to help patients with diabetes and they plan to pursue further diabetes education. This is evident in the reported patients and caregivers assisted throughout the study. Following the DSEP training, participants assisted thousands of people with diabetes and hundreds of caregivers from time points from 9 months to 3 years and 5 years (cohort 2 and cohort 1), respectively. Immediately following the program and by the end of their first year of pharmacy school, 61% and 75% (cohort 1 and cohort 2, respectively) of student pharmacists assisted both patients with diabetes and 17% and 18% (cohort 1 and cohort 2, respectively) assisted caregivers by using their knowledge in clinical settings. This difference reflects that student pharmacists were more commonly exposed to patients with diabetes compared to caregivers of people with diabetes. Remarkably, in their P1 year of pharmacy school, student pharmacists reported assisting close to 2000 people with diabetes and over 250 caregivers in both cohorts, and participants reported assisting over 17,500 patients and over 4400 caregivers over the total 3 years and 5 years of study for both cohorts. Large chain community pharmacies and clinics were consistently the most common locations, with an increase in hospital locations in later years.

While the majority of the diabetes self-care content has not changed since its inception, over the years, the DSEP training has expanded to include more active learning. In addition to using a glucose monitor for two weeks, student pharmacists concomitantly now wear a CGM for two weeks to enhance student pharmacist knowledge, empathy, and understanding of what patients experience. Students are also able to observe the impact of various foods and exercise on their own glucose values, which is also a learning opportunity. Moreover, we have also introduced a three-hour active learning workshop with pharmacist-facilitated stations including topics of self-injection administration (pen and vial/syringe), glucose monitor training, SMBG logbook glucose value interpretation, hypoglycemia prevention and treatment, glucagon education and administration, carbohydrate counting, and insulin pharmacokinetics.

The OTC Handbook of Nonprescription Drugs is a capstone guideline for pharmacy practice content in US pharmacy schools [23]. Several versions ago, the diabetes content from the OTC Handbook was completely removed from this text, along with other chronic diseases such as asthma [21]. This is unfortunate as the prevalence of diabetes continues to grow, and early detection and education have resulted in delayed progression of costly long-term complications. The results from this study demonstrate that student pharmacists can immediately apply their diabetes knowledge to assist and educate people with diabetes and their caregivers as early as the P1 year, as well as screen for diabetes. Having reached epidemic proportions, diabetes is a global concern. Based on data from the International Diabetes Federation, there are approximately 527 million adults living with diabetes, most of which have type 2 diabetes, and this value is projected to increase by 643 million by 2030 and 783 million by 2045 [2]; also concerning is the 240 million people who are yet to be diagnosed. The diagnosis of prediabetes continues to grow as well. In the US, approximately 11.6% of Americans have diabetes, and 97.6 million have prediabetes, indicating that 136 million or 41% of Americans either have diabetes or are at risk for developing diabetes [1]. Again, student pharmacists participating in community screenings can assist with diabetes prevention, early detection, and diabetes education.

Since completing the DSEP training, our student pharmacists were inspired to translate their knowledge and confidence and apply their knowledge further by creating patient-facing educational programs in our large community. These have included serving in the UC San Diego Student-Run Free Medical Clinics, which help support underserved populations [24]. They created diabetes screenings and a comprehensive foot care clinic where they collaborated with community partners to provide free footwear (socks and shoes) and diabetes supplies, a glucose monitoring clinic, and an insulin administration clinic; all student-pharmacist-run and overseen by a pharmacist faculty member.

A limiting factor inherent in any survey is the responder response rate and bias. In our study, the response rate reduced over time in both cohorts; however, the trends were similar and the study lengths of 3 years and 5 years (cohort 2 and cohort 1, respectively), were long. We attempted to limit bias by adding and comparing a second cohort. Moreover, we did not have a comparator group of non-DSEP participant pharmacists to examine the knowledge and confidence retention in diabetes self-care, yet this may be an interesting future study. Generalizability to other programs is also limited. In addition, our survey instruments, which evaluated all components of DSEP including knowledge, confidence, clinical applicability, and impact over time, were validated both externally and internally using a focus group to test and refine the survey as previously described [20]. However, further validation by the use of other research teams would be useful.

Data from this study is from a small school of pharmacy in the US, with a student body of less than 300 student pharmacists and only 60–70 first-year student pharmacists enrolled per year. Based on the American Association of Colleges of Pharmacy data, in December 2023, there were 142 colleges and schools of pharmacy with accredited professional degree programs, with 44,403 enrolled first-year student pharmacists in the US [25]. According to the International Pharmaceutical Federation (FIP), a global leader in pharmacy, they created a FIP World List of Pharmacy Schools indicating that there are close to 2000 accredited pharmacy schools worldwide [26]. Imagine the scope and impact of the DSEP training that could be incorporated into the first-year student pharmacist curriculum in all these schools across the globe.

## 5. Conclusions

An early comprehensive diabetes self-care educational program increased first-year student pharmacists’ knowledge, confidence, and skills, which they were able to apply in multiple clinical settings including community pharmacies and ambulatory care clinics. Their knowledge and application were sustained for 3 or more years following the training, providing an opportunity for them to screen and educate patients with diabetes and their caregivers. With diabetes reaching epidemic proportions, other colleges and schools of pharmacy should consider replicating early implementation of DSEP training to help increase the healthcare providers available to detect and meet the needs of the high prevalence and growing number of people with diabetes across the globe.

## Figures and Tables

**Table 1:** Pharmacy Students’ Perception of Knowledge of Diabetes and Confidence with Glucose Meters, Insulin Use, and Performing Diabetic Foot Examinations.

	Pre	Pre	Post	Post	9 Months	9 Months	1 Year	1 Year	2 Years	2 Years	3 Years	3 Years	4 Years	5 Years
Cohort	1	2	1	2	1	2	1	2	1	2	1	2	1	1
No. of Participants	59	60	59	60	59	60	52	47	26	22	18	12	4	11
Perception of Knowledge:	Average Percentage (%) Response Rating 3–5 = Confident to Excellent													
Diabetes as a disease	53	42	92	100	92	91	98	83	100	100	100	91	100	91
Risk factors for DM	55	38	95	100	92	91	96	83	100	100	100	92	100	91
Complications DM	47	34	97	100	91	81	98	87	100	100	100	92	100	90
DM1 vs. DM2	59	53	99	100	94	94	96	96	100	100	100	92	100	91
Signs and symptoms	42	32	98	100	93	93	96	88	100	100	100	92	100	90
Nutrition therapy	30	29	95	95	86	83	86	81	96	95	100	91	75	90
Exercise guidelines	36	20	94	94	93	81	90	85	92	100	100	92	75	81
Insulin therapy	22	15	93	90	75	79	73	58	96	96	94	83	100	91
Glucose monitors	24	16	95	99	92	90	88	88	92	96	83	92	75	72
Diabetic foot exams	10	2	97	98	78	83	76	67	65	95	84	92	100	54
Perception of Confidence:	Average Percentage (%) Response Rating 3–5 = Confident to Excellent													
Explaining how to use a glucose meter	25	16	100	100	95	97	96	89	97	95	88	100	100	91
Recommending a glucose meter	22	15	94	100	82	79	88	84	80	92	67	100	100	81
Performing a self-fingerstick	25	20	98	100	94	98	96	96	97	99	95	99	100	90
Performing a patient fingerstick	18	18	95	98	95	97	95	96	96	100	100	100	100	90
Teaching how to perform a fingerstick	17	16	98	100	94	96	98	93	96	96	100	100	100	81
Explaining different types of insulin	9	15	95	97	82	54	53	45	100	100	94	100	100	90
Teaching insulin injection	5	4	98	100	90	81	89	68	100	100	95	101	100	90
Discussing insulin therapies	7	6	87	97	70	52	55	54	100	101	100	100	100	90
Administering insulin injection	5	5	89	99	88	90	80	75	96	91	83	100	100	90
Giving insulin injection to patient	3	3	94	92	77	89	72	65	85	82	78	99	100	81
Teaching patients about hypoglycemia	17	13	99	100	87	83	86	85	100	100	100	101	100	90
How to treat hypoglycemia	18	10	99	100	95	87	86	84	100	100	100	100	100	90
Performing a diabetic foot exam	2	0	95	96	74	69	72	63	53	91	77	91	75	73
How to perform a diabetic foot exam	2	2	95	94	77	67	73	61	57	96	77	100	75	72
Discussing foot exams with patients	5	3	100	96	94	90	90	78	92	100	100	99	75	81
Discussing foot risk factors	3	5	100	96	90	70	88	64	84	100	94	100	75	82
Recommending non-drug foot care	3	2	79	81	73	63	62	60	69	87	56	83	50	72

**Table 2:** Performance on Diabetes Type 1 and Type 2 Case-Based Knowledge Tests.

	Pre	Pre	Post	Post	9 Months	9 Months	1 Year	1 Year	2 Years	2 Years	3 Years	3 Years	4 Years	5 Years
Cohort	1	2	1	2	1	2	1	2	1	2	1	2	1	1
No. of Participants	59	60	59	60	59	60	52	47	26	22	18	12	4	11
Case 1 Average Percentage (%) Score	46	44	94	88	88	82	87	81	95	89	96	92	97	95
Case 2 Average Percentage (%) Score	32	32	79	81	79	60	61	63	78	77	75	80	75	81

**Table 3:** Time Spent in Various Pharmacy Settings Since Participating in a Diabetes Self-Education Program.

	9 Months	9 Months	1 Year	1 Year	2 Years	2 Years	3 Years	3 Years	4 Years	5 Years
Cohort	1	2	1	2	1	2	1	2	1	1
No. of Participants	59	60	52	47	26	22	18	12	4	11
Types of Pharmacy Settings	Median Scores									
Large chain community pharmacy	3	2	3	3	3	3.5	3	5	3.5	3.5
Clinic/community pharmacy	2	2	2	2	2	3.5	3	5	3	3
Community mass-merchandise pharmacy	1	1	1	1	1	1	1	1	1	1
Inpatient hospital pharmacy	1	1	1	1	1	3	3	3	3	3
Independently owned community pharmacy, supermarket, small chain, outpatient hospital or others not listed.	1	1	1	1	1	1	1	1	1	1

The duration of time spent in setting responses to survey questions was based on a 5-point Likert scale, on which 1 was Never, 2 was ≤1 month, 3 was 2–3 months, 4 was 4–6 months, and 5 was >6 months. Scores are represented as the median.

**Table 4:** Mean Number of Times Participants Helped Diabetes Patients or Caregivers.

	9 Months	9 Months	1 Year	1 Year	2 Years	2 Years	3 Years	3 Years	4 Years	5 Years
Cohort	1	2	1	2	1	2	1	2	1	1
No. of Participants	54	48	52	47	26	22	18	12	4	11
Percentage (%) of students that had the opportunity to assist a patient with diabetes	61	75	81	83	88	91	89	100	75	90
Percentage (%) of students that had the opportunity to assist a caregiver of a patient with diabetes	17	19	42	32	65	68	89	92	75	64
How many patients with diabetes have you assisted in any way?	1295	664	3287	1162	2957	1594	2246	1814	1050	2429
How many caregivers of patients with diabetes have you assisted in any way?	118	138	907	255	787	380	460	261	475	706
How many times used overall knowledge of diabetes?	1506	691	3925	3849	3415	1745	3081	2255	1050	3550
How many times discussed with a patient the different types of diabetes?	284	216	1457	2964	936	659	606	327	120	480
How many times explained the signs and symptoms of diabetes.	621	234	1458	2942	2139	1044	1160	770	240	675
How many times discussed pre-diabetes with a patient?	680	280	1198	2329	789	308	426	350	60	332
How many times informally or formally screened a person for diabetes?	1083	665	2648	3266	1683	880	751	1050	182	710
How many times discussed the risk factors involved with diabetes?	923	449	2150	2815	1838	797	785	770	200	756
How many times make dietary recommendations to a patient with diabetes?	609	311	1881	2766	1540	713	979	1013	110	371
How many times discussed goals of therapy with a patient?	437	151	1335	2493	1429	941	903	829	235	1630
How many times provided glucose monitor education?	686	289	1818	3206	1256	543	471	555	420	435
How many times helping with glucose monitor selection?	314	88	1241	2504	303	253	317	152	190	230
How many times providing education regarding finger-stick supplies? (e.g., lancets and device)	448	156	2194	3337	729	478	722	490	804	408
How many times performing a finger stick on a patient?	967	643	2509	3226	1609	840	469	995	145	636
How many times demonstrating for a patient how to perform finger stick tests?	578	299	1923	3099	893	327	443	244	180	309
How many times discussing insulin therapy with a patient?	42	34	723	2021	1202	469	461	603	505	1573
How many times providing insulin therapy education?	55	37	648	1868	616	463	568	557	304	1438
How many times teaching a patient the proper technique for insulin administration?	61	58	629	2434	498	284	311	382	280	1234
How many times administering an insulin injection to a patient?	21	10	523	2213	36	110	41	65	6	93
How many times performing a foot exam on a diabetic patient?	13	77	326	2118	203	403	72	283	11	84
How many times explaining to a patient the importance of foot exams?	114	96	984	2509	479	514	271	444	90	363
How many times teaching a patient how to perform a foot exam?	22	62	347	2144	161	360	138	242	21	98
How many times providing foot self-care education? (e.g., risks, prevention, fungal infections).	96	94	427	2022	254	453	208	374	71	113
How many times recommending an OTC product for a foot care for a diabetic patient?	39	82	701	2023	241	339	131	96	65	108

**Table 5:** Overall Impression Following Participation in the Diabetes Self-Care Education Program.

	9 Months	9 Months	1 Year	1 Year	2 Years	2 Years	3 Years	3 Years	4 Years	5 Years
Cohort	1	2	1	2	1	2	1	2	1	1
No. of Participants	54	48	52	47	26	22	18	12	4	11
Additional DSEP Questions	Mean Scores									
Participating in the Pharmacy practice Diabetes Education Program has increased my overall knowledge of diabetes	5	4	4	4	4.5	4	5	5	5	5
Participating in the Pharmacy practice Diabetes Education Program has increased my overall confidence in helping people with diabetes.	4	4	4	4	4	4	5	5	4.5	5
Overall, I feel the Pharmacy Practice Diabetes Education Program prepared me to educate people with diabetes	4	4	4	4	4	4	5	5	4.5	5
Overall, I feel the Pharmacy Practice Diabetes Education Program prepared me to help people with diabetes	4	4	4	4	4	4	5	5	4.5	5
Overall, I feel the Pharmacy Practice Diabetes Education Program increased my interest in diabetes	4	4	4	4	4	4	4.5	5	4.5	4
I plan to pursue further diabetes education as a result of my experience in the Pharmacy Practice Diabetes Education Program	4	4	4	4	4	4	4	4	4.5	4

Response Rating Scale 1 = Not at all confident, 2 = Somewhat confident, 3 = Confident, 4 = Very confident, and 5 = Extremely confident.

## Data Availability

Data from this study are found in the corresponding tables.

## References

[1] Centers for Disease Control and Prevention National Diabetes Statistics Report Website. https://www.cdc.gov/diabetes/php/data-research/.

[2] International Diabetes Federation; Facts and Figures. https://idf.org/about-diabetes/diabetes-facts-figures/.

[3] Parker E.D., Lin J., Mahoney T., Ume N., Yang G., Gabbay R.A., ElSayed N.A., Bannuru R.R. (2024). Economic Costs of Diabetes in the U.S. in 2022. Diabetes Care.

[4] UK Prospective Diabetes Study (UKPDS) Group (1998). Intensive blood-glucose control with sulphonylureas or insulin compared with conventional treatment and risk of complications in patients with type 2 diabetes (UKPDS 33). Lancet.

[5] Nathan D.M., DCCT/EDIC Research Group (2014). The diabetes control and complications trial/epidemiology of diabetes interventions and complications study at 30 years: Overview. Diabetes Care.

[6] Lin J., Thompson T.J., Cheng Y.J., Zhuo X., Zhang P., Gregg E., Rolka D.B. (2018). Projection of the future diabetes burden in the United States through 2060. Popul. Health Metr..

[7] Nazir S.U., Hassali M.A., Saleem F., Bashir S., Aljadhey H. (2016). Association Between Diabetes-related Knowledge and Medication Adherence: Results From Cross-sectional Analysis. Altern. Ther. Health Med..

[8] Hirsch J.D., Kong N., Nguyen K.T., Cadiz C.L., Zhou C., Bajorek S.A., Bounthavong M., Morello C.M. (2021). Improved Patient-Reported Medication Adherence, Patient Satisfaction, and Glycemic Control in a Collaborative Care Pharmacist-Led Diabetes “Tune-Up” Clinic. Int. J. Environ. Res. Public. Health.

[9] Torres H.C., Pace A.E., Chaves F.F., Velasquez-Melendez G., Reis I.A. (2018). Evaluation of the effects of a diabetes educational program: A randomized clinical trial. Rev. Saude Publica..

[10] Singh R.F., Kelly P., Tam A., Bronner J., Morello C.M., Hirsch J.D. (2018). Evaluation of a short, interactive diabetes self-management program by pharmacists for type 2 diabetes. BMC Res. Notes.

[11] Badi S., Suliman S.Z., Almahdi R., Aldomah M.A., Ahmed M.H., Elkheir H.K., Ibrahim M.I.M. (2024). The Impact of Diabetes Education by Clinical Pharmacist on Quality of Life and Treatment Satisfaction of Sudanese Individuals With Type II Diabetes Mellitus: Randomized, Double-Blind, Controlled Trial. J. Prim. Care Community Health.

[12] American Diabetes Association Professional Practice Committee (2025). American Diabetes Association (ADA) Standards of Care in Diabetes—2025 is Diabetes Care. Diabetes Care.

[13] Accreditation Council for Pharmacy Education Standards 2025. https://www.acpe-accredit.org/pharmd-program-accreditation/#tab-Standards.

[14] Medina M., Stolte S., Conry J., Culhane N., Farland M.Z., Kennedy D.R., Lockman K., Malcom D.R., Mirzaian E., Vyas D. (2023). Revising the Center for the Advancement of Pharmacy Education (CAPE) Educational Outcomes and Entrustable Professional Activities (EPAs): The Report of the 2021–2022 Academic Affairs Standing Committee. Am. J. Pharm. Educ..

[15] Fazel M., Cooley J., Kurdi S., Fazel M. (2019). A co-curricular diabetes-specific elective with interprofessional students and faculty. Curr. Pharm. Teach. Learn..

[16] Westberg S.M., Bumgardner M.A., Brown M.C., Frueh J. (2010). Impact of an elective diabetes course on student pharmacists’ skills and attitudes. Am. J. Pharm. Educ..

[17] Knezevich E., Fuji K.T., Larson K., Muniz G. (2022). A Cross-Sectional Survey Study Examining the Provision of Continuous Glucose Monitoring Education in U.S. Doctor of Pharmacy Programs. Pharmacy.

[18] McIntosh T., Divine H., Taylor S. (2024). Student pharmacist’s application of the pharmacists’ patient care process during an interprofessional diabetes camp introductory pharmacy practice experience. Curr. Pharm. Teach. Learn..

[19] Ryan G.J., Foster K.T., Unterwagner W., Jia H. (2007). Impact of a diabetes certificate program on PharmD students’ knowledge and skills. Am. J. Pharm. Educ..

[20] Morello C.M., Neighbors M., Luu L., Kobayashi S., Mutrux B., Best B.M. (2013). Impact of a first-year student pharmacist diabetes self-care education program. Am. J. Pharm. Educ..

[21] Assemi M., Morello C.M., Berardi R.R., Ferreri S., Hume A.L., Kroon L.A., Newton G.D., Popovich N.G., Remington T.L., Rollins C.J. (2009). Diabetes Mellitus. Handbook of Nonprescription Drugs: An Interactive Approach to Self-Care.

[22] Cornell S., Miller D.K., Urbanski P., ACDES (2023). The Art Science of Diabetes Care Education.

[23] Handbook of Nonprescription Drugs: An Interactive Approach to Self-Care, 21st edition. https://ebusiness.pharmacist.com/PersonifyEbusiness/Shop-APhA/Product-Details/productId/362610550.

[24] Smith S.D., Marrone L., Johnson M., Gomez A., Edland S., Beck E. (2014). Clinical Outcomes of Diabetic Patients at a Student-Run Free Clinic. Fam. Med..

[25] American Association of Colleges of Pharmacy Academic Pharmacy’s Vital Statistics. https://www.aacp.org/article/academic-pharmacys-vital-statistics.

[26] International Pharmaceutical Federation, FIP World List of Pharmacy Schools. https://www.fip.org/world-list-of-pharmacy-schools.

